# Left ventricular papillary muscle rupture due to acute myocardial infarction after transcatheter aortic valve replacement

**DOI:** 10.1016/j.ijscr.2024.110637

**Published:** 2024-11-21

**Authors:** Tatsuya Horibe, Kosuke Nakata, Takafumi Hirota, Jun Takaki, Takashi Yoshinaga, Toshihiro Fukui

**Affiliations:** Department of Cardiovascular Surgery, Kumamoto University Hospital, Kumamoto, Japan

**Keywords:** Papillary rupture after TAVR, Case report, MVR, TAVR, Cardiovascular surgery

## Abstract

**Introduction:**

Mitral regurgitation is a potential complication of transcatheter aortic valve replacement. Here, we report a case of severe acute mitral regurgitation caused by papillary muscle rupture occurring 16 days after transcatheter aortic valve replacement.

**Presentation of case:**

An 82-year-old woman with severe AS was referred to our hospital. Transfemoral transcatheter aortic valve replacement was scheduled. Preoperative computed tomography revealed that the height of the left coronary artery was 8.7 cm, and a self-expandable valve was selected. The procedure was performed successfully without coronary obstruction. A complete atrioventricular block was observed on postoperative day 5, and a pacemaker was implanted. On postoperative day 13, the patient suddenly developed dyspnoea. Coronary angiography revealed stenosis of the left main coronary artery, and percutaneous coronary intervention was successfully performed. However, on postoperative day 16, she again developed sudden dyspnoea. Transoesophageal echocardiography revealed severe mitral regurgitation caused by rupture of the left ventricular papillary muscle. An emergency mitral valve replacement was performed. Her postoperative course was uneventful.

**Discussion:**

Left ventricular papillary muscle rupture due to acute myocardial infarction after transcatheter aortic valve replacement is rare. In case of anatomical risk of coronary artery occlusion after transcatheter aortic valve replacement with a self-expandable valve, careful observation for delayed coronary obstruction should be continuously performed, even when the valve is placed in a low position.

**Conclusion:**

Severe MR due to papillary muscle rupture can be a complication of TAVR. In such cases, emergency mitral valve replacement should be performed.

## Highlights

• Rupture of the left ventricular papillary muscle due to acute myocardial infarction after transcatheter aortic valve replacement is rare.
• The anatomical risk of coronary artery occlusion after TAVR is said to be in cases where the sinus of Valsalva is less than 30 mm and the height of the coronary artery is less than 10 mm.
• In case of anatomical risk of coronary artery occlusion after transcatheter aortic valve replacement with a self-expandable valve, careful observation for delayed coronary obstruction should be continuously performed, even when the valve is placed in a low position.

## 1 Introduction

Transcatheter aortic valve replacement (TAVR) is a standard treatment method for severe aortic stenosis, particularly in elderly patients. Mitral regurgitation (MR) is a potential complication that should be assessed immediately after the procedure. The impingement of the prosthetic structure on the anterior mitral leaflet due to a low implantation of the device, ruptured chordae tendineae or mitral valvular damage caused by a stiff surgical guide wire, systolic anterior motion, bundle branch block such as left ventricular conduction disturbance caused by valve implantation are the causes of MR caused by TAVR. However, we experienced a rare case of mitral valve regurgitation due to papillary muscle rupture associated with delayed coronary artery occlusion 16 days after TAVR. The work has been reported in line with the SCARE criteria [1].

## 2 Case presentation

**Fig. 1:**
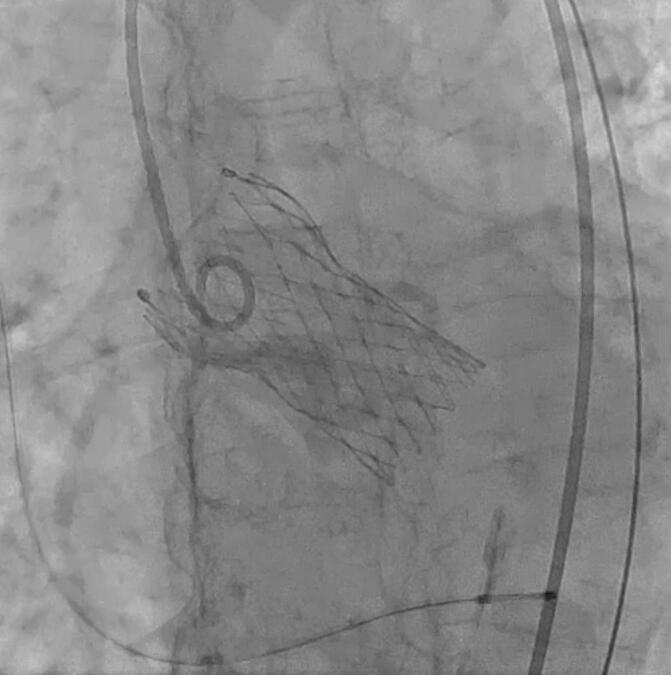
A self-expandable valve is implanted in a slightly lower position than normal. Coronary angiography shows no stenosis of the ostium of the left coronary artery.

An 82-year-old woman with severe aortic stenosis was referred to our hospital. She had shortness of breath on exertion and ejection systolic murmur. Echocardiography showed a valve area of 0.85 cm^2^, peak velocity of 4.3 m/s, mean pressure gradient of 41.6 mmHg, and preserved left ventricular ejection fraction of 60.4 % without local asynergy. Mitral regurgitation was mild without prolapse or annular dilatation. Angiography revealed no coronary artery stenosis. Computed tomography revealed that the heights of the ostia of the right and left coronary arteries were 12.1 mm and 8.7 mm, respectively. The size of the sinus of Valsalva was 29.4 × 29.0 × 28.8 mm. Our heart team thus planned transfemoral TAVR. The patient's preoperative CT showed an aortic valve calcification score of 3988 AU, indicating a highly calcified aortic valve. A self-expanding prosthesis was chosen to reduce the risk of annulus rupture and aortic dissection due to balloon expansion while taking into account the anatomical risk of coronary artery occlusion. The patient was placed under general anaesthesia and transoesophageal echocardiography was performed. The patient's hemodynamic status was stable. Implantation of a 26-mm Evolut PRO+ valve (Medtronic, Minneapolis, MN, USA) in a slightly lower position than normal and deployed via the femoral approach was accomplished (Fig. 1). Coronary angiography revealed no stenosis in the left coronary artery ostium (Fig. 1). Moreover, transesophageal echocardiography demonstrated no progression of the mitral regurgitation. Immediately after the surgery, the patient developed a complete atrioventricular block. During this time her pulse was maintained with a pacemaker, and no symptoms or findings suggested coronary artery stenosis or occlusion. Echocardiography showed EF of 65.3 %, no wall motion depression and MR showed trivial findings. On postoperative day 5, a pacemaker was implanted.

On postoperative day 12, the patient developed dyspnoea accompanied by pain in both shoulders, and elevated myocardial enzymes were observed. Echocardiography showed an EF of 38.8 %, decreased wall motion from the anterior septum to the apex, and MR showed trivial findings. However, an echocardiogram the next day showed further decreased wall motion, and MR showed more than moderate deterioration. Urgent coronary angiography revealed left main coronary artery stenosis, and direct coronary revascularization using a stent was successfully performed. Intra-aortic balloon pumping was performed to ensure hemodynamic stability.

**Fig. 2:**
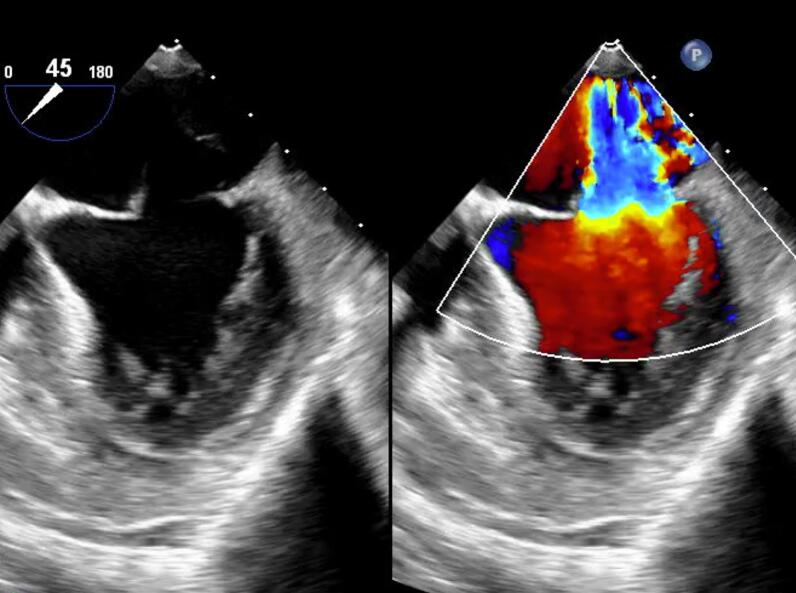
Transoesophageal echocardiography demonstrates mitral regurgitation caused by rupture of the anterior papillary muscle of the left ventricle.

**Fig. 3:**
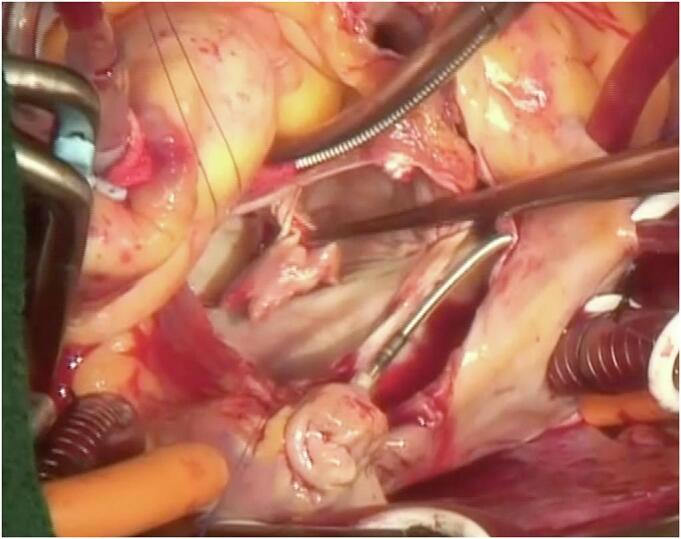
The anterior mitral leaflet with the ruptured anterior papillary muscle is resected.

**Fig. 4:**
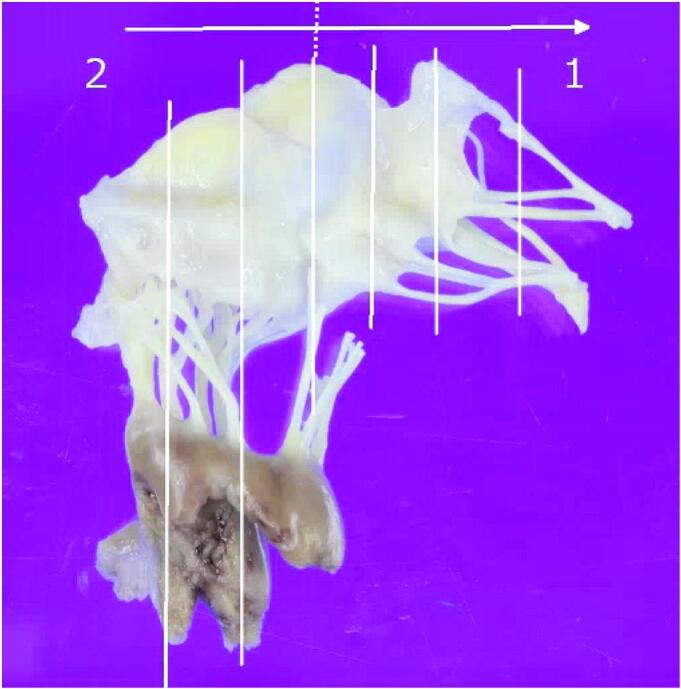
Pathological examination of the papillary muscles suggested early infarction 1 to 3 days after onset.

On postoperative day 16, she again developed sudden dyspnoea. Coronary angiography revealed a patent left main coronary artery. However, on transoesophageal echocardiography, severe MR was observed, caused by rupture of the anterior papillary muscle of the left ventricle (Fig. 2). Due to hemodynamic instability, extracorporeal membrane oxygenation was immediately established. Emergency mitral valve replacement was performed. After median sternotomy, cardiopulmonary bypass was established with ascending aortic arterial cannulation and bicaval venous cannulation. After cardiac arrest, the left atrium was opened using a transseptal approach. The anterior mitral leaflet with the ruptured anterior papillary muscle was resected (Fig. 3). A tissue valve was implanted in the mitral position without any complications on the TAVR valve. After weaning from cardiopulmonary bypass, extracorporeal membrane oxygenation was no longer necessary. The operative, cardiopulmonary bypass, and aortic clamping times were 200, 79, and 56 min, respectively. The patient's postoperative course was uneventful, and she was transferred to the ward after 7 days. Pathological examination of the papillary muscles suggested early infarction 1 to 3 days after onset, and the results indicated that papillary muscle rupture associated with infarction was suspected (Fig. 4).

## 3 Discussion

Rupture of the left ventricular papillary muscle due to acute myocardial infarction after transcatheter aortic valve replacement is rare.

In a multicentre registry, symptomatic coronary obstruction following TAVR was observed in 0.66 % of the patients [2]. Older age, female sex, no previous coronary artery bypass graft, use of a balloon-expandable valve, and previous surgical aortic bioprosthesis were risk factors for coronary obstruction. Moreover, a low-lying coronary ostium and a shallow sinus of Valsalva were associated anatomical factors. Once coronary obstruction occurred, 30-day mortality rate increased to 40.9 %. Another large international multicentre registry demonstrated an overall in-hospital death rate of 50 %, which increased if coronary obstruction occurred ≤7 days from the index procedure [3]. An anatomical risk of coronary artery occlusion after TAVR is considered in cases in which the sinus of Valsalva is <30 mm and the height of the coronary artery is <10 mm. In the present case, the sinus of Valsalva was <30 mm, and the height of the left coronary artery orifice was 8.7 mm. As the risk for coronary obstruction was considered to be high, careful heart team discussions led to the decision to use a self-expandable valve placed in a slightly lower position than normal and a guide wire was inserted into the coronary artery to prevent occlusion of the coronary artery. This case suggests that the occurrence of delayed coronary obstruction should be continuously monitored after using a self-expandable valve, even when it is placed in a low position.

MR is a complication that may occur during and after TAVR, which may require different treatments, depending on the mechanism. López-Aguilera et al. reported that 8.5 % of patients with self-expandable valves developed severe MR during the procedure [4]. Several reasons for MR have been reported, including: 1) mechanical asynchrony owing to a new left bundle branch block; 2) aortic prosthesis impingement on the anterior mitral leaflet; 3) the occurrence of systolic anterior movement of the anterior mitral leaflet with dynamic obstruction of the left ventricular outflow tract; 4) commissural tearing of the valve; and 5) a “functional” mechanism, probably due to transient damage of the subvalvular mitral apparatus by the delivery system [4,5]. Entanglement of the guidewire in the papillary muscles and extension of the guidewire into the left atrium have likewise been reported as reasons for the development of MR [6]. Based on these anatomical risks, the type of bioprosthetic valve to be selected and the approach method are determined, and we can predict complications that may occur after surgery.

There have been no reports of delayed postoperative worsening of MR due to stenosis of the left main coronary artery. In our case, although the left main coronary artery was successfully recanalized by urgent coronary stenting, damage to the left ventricle, including the papillary muscle, was severe. Intraoperative findings showed no obvious interference of the bioprosthetic valve to the mitral valve and no damage to the mitral valve leaflets. Histopathological findings also showed findings suggestive of acute infarction in the papillary muscle, and it was centrally suggested that papillary muscle rupture due to myocardial infarction was the cause of the sudden severe MR after TAVR. During surgery, there is a possibility that the aortic bioprosthetic valve may become deformed or displaced. If the aortic valve was deformed due to surgery and AR or AS occurred, we would perform AVR in addition to MVR. Therefore, when weaning the patient from cardiopulmonary bypass, we observed not only the mitral valve but also the aortic valve and proceeded with the surgery after confirming that the aortic bioprosthetic valve was not damaged, deformed, or displaced. This seems to be a particularly important point in cardiac surgery performed in the acute phase after TAVR.

## 4 Conclusion

Severe MR due to papillary muscle rupture can be a complication of TAVR. In such cases, emergency mitral valve replacement should be performed. The anatomical risk of coronary artery occlusion after TAVR is said to be in cases where the sinus of Valsalva is <30 mm and the height of the coronary artery is <10 mm. Patients at high risk may develop delayed coronary artery occlusion, so careful post-operative care is required.

## Author contribution

Tatsuya Horibe - writing the paper, data collection.

Kosuke Nakata - data collection.

Takafumi Hirota - data collection.

Jun Takaki - paper review.

Takashi Yoshinaga - paper review.

Toshihiro Fukui - writing the paper, paper review.

## Consent

Written informed consent was obtained from the patient to publish this case report and accompanying images. Patients can share their views on the treatment they have received if appropriate. A copy of the written consent is available for review by the Editor-in-Chief of this journal on request.

## Ethical approval

This article does not contain any personal information that can lead to the identification of the patient. My institution does not require ethical approval regarding case reports per local policy.

## Guarantor

Tatsuya Horibe.

## Funding

None.

## Conflict of interest statement

None.
